# Behavioral responses of blue-winged teal and northern shoveler to unmanned aerial vehicle surveys

**DOI:** 10.1371/journal.pone.0262393

**Published:** 2022-01-19

**Authors:** Mason D. Ryckman, Kaylan Kemink, Christopher J. Felege, Brian Darby, Gregory S. Vandeberg, Susan N. Ellis-Felege

**Affiliations:** 1 Department of Biology, University of North Dakota, Grand Forks, ND, United States of America; 2 Ducks Unlimited, Inc., Bismarck, ND, United States of America; 3 Geography Department, University of North Dakota, Grand Forks, ND, United States of America; Texas State University, UNITED STATES

## Abstract

Unmanned aerial vehicles (UAVs) have become a popular wildlife survey tool. Most research has focused on detecting wildlife using UAVs with less known about behavioral responses. We compared the behavioral responses of breeding blue-winged teal (*Spatula discors*) (n = 151) and northern shovelers (*Spatula clypeata*) (n = 46) on wetlands flown over with a rotary DJI Matrice 200 quadcopter and control wetlands without flights. Using a GoPro camera affixed to a spotting scope, we conducted focal individual surveys and recorded duck behaviors for 30 minutes before, during, and 30 minutes after UAV flights to determine if ducks flushed or changed in specific activities. We also conducted scan surveys during flights to examine flushing and movement on the entire wetland. Between 24 April and 27 May 2020, we conducted 42 paired (control and flown) surveys. Both teal and shovelers increased proportion of time engaged in overhead vigilance on flown wetlands from pre-flight to during flight (0.008 to 0.020 and 0.006 to 0.032 of observation time, respectively). Both species left the wetland more frequently during flights than ducks on control wetlands. Despite similarities between species, we observed marked differences in time each species spent on active (e.g., feeding, courtship, swimming), resting, and vigilant behaviors during flights. Overall, teal became less active during flights (0.897 to 0.834 of time) while shovelers became more active during this period (0.724 to 0.906 of time). Based upon scan surveys, ducks flushed in 38.1% of surveys while control wetlands only had a single (2.4%) flush during the flight time. We found launch distance was the most important predictor of whether ducks swam for cover or away from the UAV which could result in inaccurate counts. Ducks appear aware of UAVs during flights, but minimal behavioral shifts suggest negative fitness consequences are unlikely.

## Introduction

Annual breeding bird population surveys are critical for understanding productivity, establishing conservation priorities, and setting harvest regulations for some species. Decision-making associated with these surveys requires having reliable and consistent data collection efforts that often employ low-flying aerial surveys or conducting intensive ground surveys. Aerial surveys can be prohibitively expensive and even dangerous to conduct. In fact, due to the low altitude and slow speeds, occupied aerial surveys accounted for 66% of job related mortality in wildlife researchers from 1937–2000 [1]. Further, ground surveys can be labor intensive, inconsistent due to observer variation, and logistically difficult in remote areas [2–4]. As a result, wildlife professionals and scientists are increasingly interested in using unmanned aerial vehicles (UAVs) for surveys because of the safe and cost-effective alternatives they provide to traditional aerial surveys [5, 6].

UAVs confer numerous advantages both in and out of the field for wildlife professionals. Using this technology, researchers can access terrain which would otherwise be inaccessible to ground observers. Their use can also help address the challenge of observer fatigue, which can bias the data being collected [7, 8]. Outside of fieldwork, UAVs allow researchers to create an archive of data, giving researchers the chance to return to a dataset for multiple observers to verify estimates or even explore questions that may arise in the future [9]. Also, UAVs provide high resolution imagery that can overcome problems of timing (e.g., plant and breeding season phenology) and suboptimal atmospheric conditions (e.g., cloud cover) that often hinder traditional satellite-based remotely sensed data [4]. Finally, one of the most cited benefits of UAVs is the reduced anthropogenic disturbance they provide compared to other survey techniques [10, 11]. However, wildlife responses are often difficult to evaluate and until recently the benefit of reduced disturbance was based on anecdotal evidence. Advances in this field have demonstrated that responses to UAV disturbance are species-specific [9, 11] and that birds might exhibit higher sensitivity to UAVs than other wildlife [11].

While the “birds eye view” captured by UAVs may help reduce bias in surveys, this benefit is contingent on the UAVs not disturbing the wildlife in question. Determining whether UAVs elicit negative behavioral responses such as escaping or leaving the area is important as these behaviors can reduce the time spent on fitness-enhancing activities such as feeding or mating [12]. Negative behavioral responses may also influence survey results if the individuals leave or move because of the survey method, causing a missed detection or double-count. Some studies have shown UAVs are capable of adversely impacting wildlife species by allowing aerial predators to depredate nests following a UAV flight or causing stress by increasing heart rates in individuals [13, 14]. As Mulero-Pazmany et al. (2017) suggest, responses are often species-specific and depend on a variety of characteristics of the animal, landscape topography, and the UAV platform. Careful evaluation of life-history stage and level of aggregation of a species need to be evaluated.

One group of wildlife UAVs are being used to survey more frequently is waterfowl [9, 15, 16]. Traditional surveys, such as the Waterfowl Breeding Population and Habitat Survey (WBPHS), are conducted via low-flying airplanes. The WBPHS surveys most of the North American breeding duck population and has been conducted annually since 1955 using a fixed-wing aircraft flying at an altitude of 30-50m above the ground. UAVs have been used thus far to survey nesting or breeding waterfowl [16, 17] and also nonbreeding waterfowl [9, 18–20]. The emphasis of UAV use in waterfowl research not only parallels the broader wildlife arena in its rapid growth but also in its limited information on behavioral responses of breeding ducks with only a few studies to some extent examining behavioral responses [9, 17, 20]

Here, we tested the assumption that UAVs do not alter behaviors such as vigilance and escape (e.g., flush, move to cover) by flying a rotary UAV over wetlands using altitudes and flight patterns that would be used to conduct actual breeding pair surveys. Specifically, we used a before-after-control-impact design to determine if breeding dabbling ducks flush, move to adjacent cover, or change in specific activities when flown over, and these flights were compared to control wetlands (no UAV flights) starting before flight and monitoring behaviors through post-flight period. From this research, we suggest guidelines for wildlife researchers conducting waterfowl surveys with UAVs that describe limitations of UAVs and illustrate approaches that minimize shifts in behavior responses while maximizing the quality of data generated.

## Methods

### Study area and study species

Breeding duck surveys took place in the Prairie Pothole Region of North Dakota at two ranches: Coteau Ranch and Davis Ranch located in Sheridan County. Operations were based out of a location on the Coteau Ranch (N 47.401054, W -100.276947). The Coteau Ranch is currently owned by Ducks Unlimited and is approximately 1,214 ha. The Nature Conservancy owns the Davis Ranch which is approximately 2,931 ha. The ephemeral and semi-permanent wetlands of the area attract a variety of dabbling duck species to the study area. We chose to focus on northern shoveler (*Spatula clypeata*) and blue-winged teal (*Spatula discors*), which were the most common species present on wetlands within our survey area.

### Behavior monitoring and point counts

We conducted UAV flights over wetlands that had ducks present at the start of the entire survey and remained on the wetland through the first observation period. For each trial, we collected behavior data and duck counts at paired wetlands: a control and a flown. We defined a control wetland as a ponded wetland we identified prior to UAV flights that had ducks present and that we did not fly a UAV over or within 115 meters of, and it did not have a previous UAV flight on it. We acknowledge that depending upon wind and other weather characteristics, the sounds of setting up equipment or the UAV in flight may be able to be detected at a control wetland. Flown wetlands were those ponded wetlands that also had ducks present, and we flew over with a UAV to obtain counts. We did not fly over the same wetland twice; however, some control wetlands later became flown. Observers collected behavior data 30 minutes prior to the UAV flight, during flight (range: 6–29 minutes) and 30 minutes after the flight. During each flight, one observer was located on a flown wetland and the other on a control wetland. Both observers sat or kneeled in cover at vantage points where the whole wetland was visible or if the wetland exceeded UAV capabilities, the observer positioned themselves where they could see only the survey area covered by the flight. Observers selected the species based on the same species being present at the paired location. If multiple individuals of a species were present, observers prioritized pairs to be able to capture both male and females and maximize sample size. Due to the presence of pairs (male and female ducks in close proximity to one another), observers were commonly able to record observations for both individuals (pair). If multiple pairs existed, observers randomly selected a pair. These pairs are described hereafter as focal individuals or focal individual if only one duck was present on the wetland [21]. Observers recorded observations of focal individuals using a GoPro Hero 4 mounted to either a Swarovski (STS 65) or Leica (APO-TELEVID 77) spotting scope. Each spotting scope was attached to a tripod (Zomei Q111, Cabelas 364/MG10) and each GoPro was attached to the spotting scope by using a phone scope attachment (C3-099-A, C3-022-1). GoPros were equipped with 16GB micro SD cards. Observation start and end times were coordinated via text messages among the two wetland observers and the UAV operator.

### Flight operations

The UAV operator conducted flights using a DJI Matrice 200 V2 (color: black, weight: 4.53kg, operating temp: -20°C to 40°C), a quad-rotary aircraft powered by lithium polymer batteries (22.8V, 7660 mAh). A Zenmuse X5S camera (RGB) was attached to the UAV. An Olympus lens with focal length of 45mm was attached to the camera. With this lens, the sensor has a ground sampling distance (GSD) of roughly 0.44cm per pixel at 45 m (150ft) above ground level (AGL). We chose 45m due to test flights that allowed pixel sizes (GSD of 0.44cm) capable for us to accurately identify ducks to species and sex. The camera was set to take still images to be used for counts, but these images were not used for any of the behavioral assessments. While counts are not reported, we report sensor parameters to contextualize actual survey parameters since we desired evaluating behaviors following actual breeding pair survey characteristics. The UAV operator preprogrammed flights using the DJI Pilot software (version 1.8.0). The flight path was a tangential approach grid (lawn-mower pattern). Research suggests this approach causes less disturbance than directly approaching birds from takeoff [9]. The UAV operator flew the UAV at 5m/s which allowed us to collect data over larger wetlands compared to slower speeds, but still obtain images that were not blurry. Flight time with this aircraft is limited to 38 minutes or less so the UAV operator flew the UAV with a 60% forward and side-to-side overlap between adjacent images to allow for orthomosaics to be produced in future studies. Full details of flight operations and conditions are reported following Barnas et al. 2020 (S1 File). Permissions were provided by the North Dakota Game and Fish Department (GNF04912726, GNF05182785), UND Institutional Animal Care Use Committee A3917-01, Protocol #1904–2, and the UND Unmanned Aircraft Systems Research Compliance Committee Approval (Approved April 12, 2019).

### Video review and individual behavioral classifications from focal surveys

We retrieved micro-SD cards from the ground observer’s GoPros after each flight and downloaded video files to a hard drive at the end of each survey day. To provide consistent behavioral evaluations, a single observer reviewed all video using Windows Media Player (Microsoft, Seattle, WA). This observer (MR) matched video files with start of survey times and classified behaviors from 30 minutes prior to takeoff until 30 minutes after the UAV has landed. The pre-flight period provided a baseline for behavioral comparisons that were individual or wetland specific. The post-period assessment provided an opportunity to determine if any residual behavior responses persisted after landing.

The observer classified behaviors of focal individuals into 5 broad categories: active, none, vigilant, overhead vigilance, and flush (Table 1). Active consisted of behaviors including preening, feeding, breeding (copulation, courtship displays), and swimming. The category none consisted of sleeping or resting behaviors. Head popping (described as high scan by Barnas et al. (2018)) was the additional behavior for vigilant and described occasions when the duck extended its head away from the body to scan its surroundings. Overhead vigilance (classified as head cocking by Barnas et al. (2018)) was when a duck tilted its head to see what was up above it. Flush consisted of either territorial flushes or other flushes for which the cause was unknown, but the bird left the wetland.

**Table 1:** Categories and types of behaviors for focal behavior surveys on breeding duck pairs conducted in Sheridan County, North Dakota during the spring of 2020.

Categories	Additional Behaviors
Active	Preening, Feeding, Breeding, Swimming
None	Sleeping, Resting
Vigilant	Head Popped
Overhead Vigilance	Head Cocked/tilted
Flush	Flush territorial, Flush other, Offshore flush

### Behavioral classification from scan surveys

In addition to focal individual surveys, we conducted scan surveys where ground observers at both flown and control wetlands recorded if birds flushed at any time during the UAV flight. Similarly, the ground observers also recorded if ducks swam towards cover or away from the UAV. This gave us the opportunity to determine bird responses to UAV flights on a wetland perspective. Specifically, we were interested in determining if treatment (flown or control), wetland characteristics (cover type class as defined by Stewart and Kantrud (1971)), proximity of flight launch site or weather characteristics (wind speed, wind direction, temperature, and cloud cover) impacted responses of ducks across the wetland. Weather data (wind speed, temperature) was collected using Kestrel 3000 at 2 points during flight (start and end) and averaged to obtain one value.

### Data analysis

#### Focal individual responses

Our analysis of focal individual responses used a before-after-control-impact (BACI) design. In the model set, we looked at proportion of time spent in each of the five pre-defined behaviors. We constructed a generalized mixed model using Proc Glimmix in SAS Studio (Version: 9.4) assuming that each of our five behaviors (active, none, vigilant, overhead vigilance, flush) were drawn from a beta distribution representing the proportion of time spent in that behavior. Fixed effects included *species* (blue-winged teal, northern shoveler), *treatment* (flown or control), *flight period* (before, during, after), the 2-way interactions of *treatment* and *flight period*, *species* and *flight period*, and the 3-way interaction of *species*, *treatment*, and *flight period*. For a significant effect in a BACI design, we would expect to see a significant interaction between treatment and flight period. Random effects were *flight ID* and *bird ID*(species). P-values < 0.05 were considered statistically significant. We calculated the least square means (LSMeans) and report these estimates and their associated 95% confidence intervals.

#### Behavioral responses from scan surveys

To evaluate general observations of ducks across the wetlands, we used a Fisher Exact test to compare flush levels (none, <50% of all birds flush, 50% - 99% of all birds flush, all individuals flush) for the two categories of treatment (wetland was flown over) and control (wetland did not have a flight). We considered P-values < 0.05 to be significant and provide summary statistics for each flush level by treatment (flown, control).

For our scan surveys, we wanted to know if birds might be moving away from the UAV or towards cover during surveys and if other factors may play a role in responses. We constructed a logistic regression using Proc Glimmix is SAS Studio (Version: 9.4) to model the binary response of ducks swimming away from UAV or to cover (1) versus no visible movement as ducks stayed in the relative same area throughout the flight period (0). Variables used in this model were *treatment* (flown or control), average *temperature* during survey time, average *wind* speed during survey, *cloud cover* (clear, partly cloudy, mostly cloudy, and overcast), *wind direction* (8 categories of N, S, E, W, NE, NW, SE, SW), wetland *cover type* [22], UAV *survey size* (area coverage of programmed flight) to represent wetland size, and *launch distance* (Euclidean distance of edge of wetland to UAV launch site). We included wind parameters because the UAV might have a higher noise output with high winds and birds might respond differently [23]. To explore if ducks responded differently to shadows produced from the UAV, we included cloud cover in the model. By including launch distance, we assessed whether the anthropogenic influence of the UAV launch operation is impacting how birds are behaving [10]. We included cover type and survey area of the wetland because emergent vegetation and relative size could influence how birds respond to UAV flights. We used Akaike Information Criterion corrected for small samples sizes (AICc) to evaluate the simplest explanation for if birds swim to cover [24]. We calculated odds ratios from back transforming estimates to present how many times more or less likely the probability of swimming away was relative to the covariate of interest.

## Results

We collected behavioral observations on control and flown wetlands for 42 flights between April 24^th^ and May 27^th^, 2020. In a few cases (n = 3), technological failures, observer errors, or external disruptions (e.g. rancher drove ATV next to control wetland flushing all birds) rendered insufficient data for analysis. Initially, we obtained behavioral observations for 7 species of ducks, but due to difficulty in collecting adequate samples sizes of each, we focused efforts exclusively on blue-winged teal and northern shoveler. This translated to behavioral observations from 32 flights on 151 blue-winged teal and 13 flights on 46 northern shovelers. On average, we monitored 2.6 focal indivduals/ flown wetland and 2.7 focal individuals/ control wetland. Average UAV flight duration was 18 min (range: 6–29 minutes).

### Individual duck behavioral response to UAV flight

**Fig 1:**
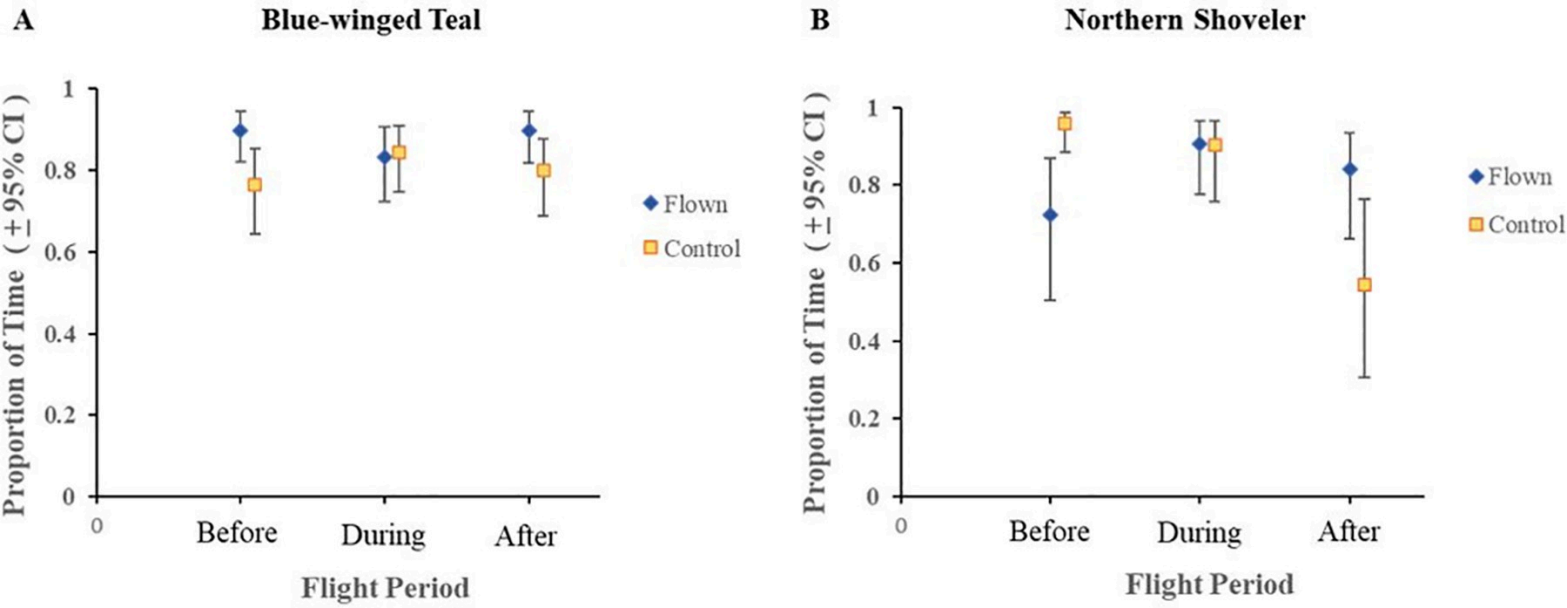
Least square means and 95% confidence intervals of proportion of time spent on active behavior for blue-winged teal (A) and northern shoveler (B) within treatments groups (Flown vs. Control). Additional behaviors for active are feeding, breeding, preening, and swimming.

**Fig 2:**
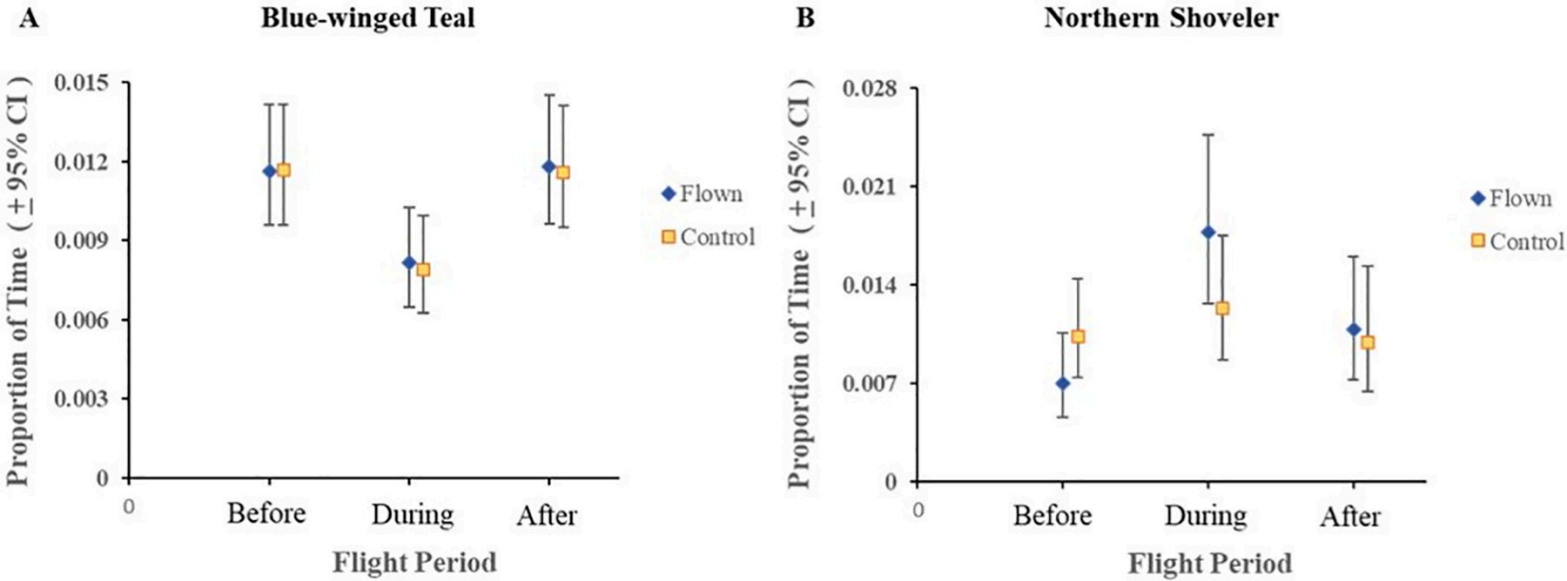
Least square means and 95% confidence intervals of proportion of time spent on vigilant behavior for blue-winged teal (A) and northern shoveler (B) within treatments groups (Flown vs. Control). This behavior consisted of the ducks fully extending their head away from the body to scan for intruders.

**Fig 3:**
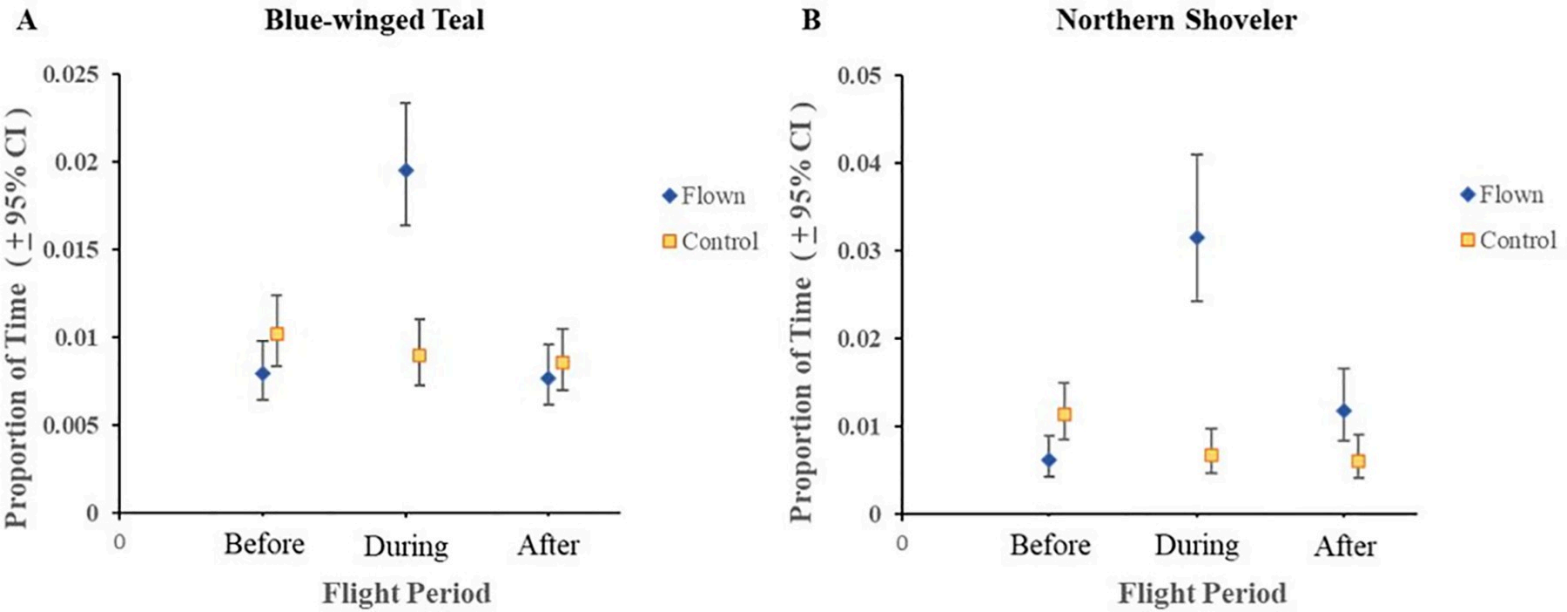
Least square means and 95% confidence intervals of proportion of time spent on overhead vigilance behavior for blue-winged teal (A) and northern shoveler (B) within treatments groups (Flown vs. Control). This behavior consisted of the ducks tilting their head to look at the UAV above them.

For active and none (sleeping or resting) behaviors, we found a statistically significant difference in behaviors (P < 0.05) for flight period and all the interactions (treatment × flight period, species × flight period, species × treatment × flight period; Table 2). Proportion of time spent on active behavior for blue-winged teal on flown wetlands decreased from pre-flight period when the UAV was overhead (0.897 to 0.834; Fig 1A; S1A Table). In other words, for a 10 minute observation period teal spent almost 9 minutes on average engaged in active behaviors prior to the flight, but reduced time spent in that behavior to 8.34 minutes during a 10-minute flight. In contrast, active behavior for northern shovelers on flown wetlands increased from pre-flight period when the UAV was overhead (0.724 to 0.906; Fig 1B). For a 10-minute pre- and during flight observation period, shovelers increased activity from 7.24 minutes to over 9 minutes during the flight. For both species, post-flight responses were similar to pre-flight responses (Fig 1). For vigilant behavior, the two-way interactions of treatment × flight period and species × flight period were statistically significant (Table 2). Vigilant behavior for blue-winged teal decreased from pre-flight period to when the UAV was overhead (0.012 to 0.008; Fig 2A) while northern shovelers increased from the pre-flight period to when the UAV was overhead (0.007 to 0.018%; Fig 2B). This translated to teal spending approximately 7 seconds in vigilant behaviors during a 10-minute pre-flight observation to less 5 seconds during a 10-minute flight, while shovelers increased vigilance from about 4 seconds during the pre-flight period to over 10 seconds in vigilant behavior during a 10-minute flight period. Similar to active behaviors, vigilance appeared to return to similar pre-flight levels (Fig 2). We found statistically significant results for treatment, flight period, the two-way interaction of treatment × flight period, and the three-way interaction of species × treatment × flight period for the proportion of time spent in overhead vigilance behavior (Table 2). Blue-winged teal and northern shovelers both exhibited increases in overhead vigilance from pre-flight period to when the UAV was overhead (0.008 to 0.020 and 0.006 to 0.032), but these levels returned to pre-flight levels during the post-flight period (Fig 3).

**Table 2:** Model results for proportion of time spent on each behavior. Fixed effects include species (blue-winged teal and northern shoveler), treatment (flown and control), flight period (before during after), and the interactions (treatment × flight period, species × flight period, species × treatment × flight period). Bold highlights denote statistical significance.

Behavior	DF	F-value	P-value
**Active**			
species	1	0.02	0.9011
treatment	1	0.35	0.5525
** flight period**	**2**	**5.73**	**0.0037**
**treatment** × **flight period**	**2**	**10.55**	**<0.0001**
**species** × **flight period**	**2**	**8.24**	**0.0003**
**species** × **treatment** × **flight period**	**3**	**10.00**	**<0.0001**
**None**			
species	1	0.01	0.9146
treatment	1	1.08	0.2989
** flight period**	**2**	**7.14**	**0.0010**
**treatment** × **flight period**	**2**	**11.34**	**<0.0001**
**species** × **flight period**	**2**	**10.02**	**<0.0001**
**species** × **treatment** × **flight period**	**3**	**11.13**	**<0.0001**
**Vigilant**			
species	1	0.25	0.6184
treatment	1	0.02	0.8787
flight period	2	0.85	0.4279
**treatment** × **flight period**	**2**	**3.08**	**0.0475**
**species** × **flight period**	**2**	**19.00**	**<0.0001**
species × treatment × flight period	3	1.70	0.1664
**Overhead Vigilance**			
species	1	0.03	0.8683
** treatment**	**1**	**12.95**	**0.0004**
** flight period**	**2**	**32.02**	**<0.0001**
**treatment** × **flight period**	**2**	**63.83**	**<0.0001**
species × flight period	**2**	0.80	0.4515
**species** × **treatment** × **flight period**	**3**	**7.59**	**<0.0001**

### Scan surveys behavioral responses

We observed more events of ducks flushing on flown wetlands than at control wetlands (Table 3). For all observations when all ducks flushed on the flown (n = 2 flights) and control wetlands (n = 1 flight), there was only 1 pair of ducks on the wetland at the time of the flight. Results from the Fisher’s Exact test indicated there was a significant difference between flush levels for wetlands that were flown over and wetlands that were not flown (p-value < 0.01).

**Table 3:** Summary statistics for a multinomial classification of flushes on a whole wetland perspective basis. We conducted a total of 42 flights during the spring and early summer of 2020.

Flush Category	Flown	Control	Total
None Flushed	26	41	67
< 50% Flushed	12	0	12
50–99% Flushed	2	0	2
All Flushed	2	1	3
Total	**42**	**42**	**84**

Based on summary statistics, if birds were to swim away from the UAV or into cover, this occurred more often on flown wetlands (Table 4). However, the most parsimonious model (lowest AICc score) explaining the binary response of ducks swimming towards cover during surveys was the additive model of intercept + launch distance (distance from edge of wetland to UAV lauch site: S1b Table). This model possessed >98% AICc weight. Based on our top model, we found the probability of ducks swimming away from the UAV or towards cover was about 1.4 times less (OR = exp(-0.003*100)) likely for each 100 m increase in distance between the edge of wetland and the launch site (Intercept = 0.535, SE = 0.613; *B*_*distance from pad*_ = -0.003, SE = 0.002).

**Table 4:** Summary statistics for the binary response of birds swimming away or towards cover during the UAV flight on a whole wetland perspective basis. We conducted a total of 42 flights during the spring and early summer of 2020.

Swam Description	Flown	Control	Total
Did not swim away or towards cover	23	36	59
Swam away/ towards cover	19	6	25
Total	**42**	**42**	**84**

## Discussion

With an increased interest in quantifying behavioral responses of wildlife to UAVs, our study is the most detailed and systematic behavioral evaluation of breeding ducks relative to a multi-rotor UAV. Our flight methods followed procedures that researchers would use to conduct breeding pair surveys using UAVs. We used a BACI design to evaluate changes in behaviors of focal individuals. We observed a quantifiable behavioral change on flown wetlands for blue-winged teal and northern shovelers across the flight periods (before, during, after). On flown wetlands, the proportion of time spent on overhead vigilance increased during the flight period and decreased back to pre-flight levels during the post-flight period for both species. This finding suggests both species of ducks were detecting the aircraft above them. These results are similar to a study looking at behavior changes of lesser snow geese (*Anser caerulescens caerlescens*) to UAV flights [8] and also noted in other waterfowl species surveyed with UAV models [9]. Further, other species of Antarctic birds have been documented engaging in aerial vigilance in response to UAVs [25, 26]. As Barnas et al. (2018) noted, the time spent in this behavior overall is a very small proportion of total time and therefore unlikely to have a large biological impact. We agree that for the ducks we surveyed, it is interesting that they notice the UAV in flight like other species of wildlife, but that this should not limit the use of UAVs as a sampling tool. Further, many other survey approaches such as observers approaching a wetland on foot or manned aviation likely draw the same vigilance response if not more since the birds may flush.

While active behaviors (swimming, breeding, feeding, preening) decreased a small amount for blue-winged teal during the flight, northern shovelers had an increase in active behavior. We acknowledge that we were unable to disentangle in focal birds if the swimming was suggesting a disturbance type response or simply just swimming as ducks do and that these were small proportions of time relatively speaking. However, it is worth noting that some birds do tend to become more active in response to UAVs such as those that are generally being disturbed by UAVs [27], while others tend to hold still [28]. Thus, it can be difficult to discern the motivation of an active behavior such as swimming. Further, we saw similar patterns for the proportion of time spent on vigilant behavior (head is extended away from body) for both species. Interestingly, both flown birds and control birds had the same pattern suggesting birds may be using their auditory stimuli to detect the aircraft. Blue-winged teal might be decreasing active and vigilant behavior as an anti-predatory response to the perceived threat of the UAV as a predator [12]. Perhaps most importantly, our findings demonstrate that during the post-flight period, ducks were resuming pre-flight levels of the behaviors, meaning these shifts in behavior were not sustained after the UAV landed. The species-specific responses in activity and vigilance were interesting given the two species are similar in size, and likely would have similar risks from aerial predators. However, it is worth noting that the two species have different life history patterns in diet and foraging ecology (shovelers are more filter feeders compared to teal) and likely different harvest pressure with teal being a more sought after game species than shovelers that may impact responses to potential threats.

During our scan surveys where we explored flushing of birds across the wetland, we found that ducks more often flushed on flown wetlands compared to control wetlands. Although the instances where all ducks flushed were few, this only occurred when the number of ducks on the wetlands (flown = 2, control = 1) were a lone pair. While we didn’t explore group size in detail and this warrants further investigation, our results from anecdotal evidence suggest ducks are more likely flush in response to UAVs on small wetlands with few ducks. This contrasts with past research that noted larger groups of ducks tend to flush during UAV surveys compared to smaller group sizes [11].

We also found from our scan surveys, that ducks were more likely to swim towards cover or away from the UAV during the flight on flown wetlands. While that behavior was also apparent on control wetlands (14%), there are a few possible explanations for our model results. First, due to UAV battery life limiting flight durations and to reduce the proximity of the UAV to a control wetland, we set up launch sites closer to flown wetlands (average = 319 m) than control sites (average = 537 m). Second, birds are likely responding to disturbance caused by researchers setting up equipment for UAV surveys which could lead to responses based on proximity to launch site. This has also been noted in past research of waterfowl species [8, 10]. Barnas et al. (2018) reported that if snow geese were to leave their nest, it most often occurred before the UAV was even launched. Vas et al. (2015) recommended launch sites be greater than 100 meters from the wetland to minimize disturbances. Given most of our launch sites were greater than 100 m and averaged 428.18 m, we suggest even greater distances from launch sites to wetland may be warranted.

When examining the overall findings from this work, we did observe behavioral shifts in ducks reacting to UAV but responses were minimal in overall proportion of time and relatively rapid returns to pre-flight behaviors. These behavioral responses were unlikely to have major fitness consequences on breeding ducks and should not dissuade their use as a potential tool for breeding pair surveys. However, it is unknown if repeated flights over wetlands may result in habituation or increased disturbances that could have negative implications for breeding productivity. If birds are constantly flushed or increasing movement with avoidance behaviors such as swimming to cover, this could have energetic costs [29]. Current survey protocols for breeding waterfowl typically have limited visits (two per survey period) so this is unlikely to be a major concern, but if other questions or monitoring efforts require intense, repeated surveys, this should be considered.

Our study was designed to evaluate behavioral responses that would mimic a survey for estimating breeding pairs. As a result, we used a sensor that enabled us to be at 45 m (150 ft) AGL. Many off-the-shelf cameras may not have this resolution and result in a need to fly at a lower altitude. In fact, much of the research to date has been conducted at very low altitudes (e.g. 31m, 40m) in order to obtain accurate information on breeding birds [9, 16]. We highly encourage consideration of sensors and UAV platforms that maximize altitude while still meeting image resolution needs [8, 30]. Thus, altitudes lower than 45 m may result in very different behavioral responses than we found in our study.

The primary motivation of this work was to understand if we were to adopt UAVs as a tool for breeding pair surveys, are UAVs going to negatively impact breeding birds and would changes in behavior cause challenges in obtaining accurate counts. Our work suggested that UAV flights can cause flushes and this may result in missing ducks in counts compared to ground counts where an observer can take note of this in the field if it were to occur. We found ducks swam away from the incoming UAV, possibly perceiving it as a threat, and such movements can result in double counting or missing ducks entirely during a survey. Further, ducks moving away from the UAV or towards cover may reduce their ability to be detected depending on the type and resolution of the sensor. However, ground observers may also move birds or be unable to detect birds for these same reasons, thus creating some similar detection challenges. The benefit of a UAV is the ability to attach different sensors such as dual sensors equipped with a Red, Green, Blue (RGB; also known as an electrical optical sensor) and thermal or potentially even ultraviolet sensor on a UAV platform. This may aid in improving the detection of ducks in vegetation or along wetland edges that would be missed otherwise when using just an RGB camera (Helvey 2020). Technological advances in sensors and automation of imagery to obtain accurate counts will be critical next steps in the adoption of UAVs for duck counts [31, 32]. Furthermore, future research is needed to understand the actual counts obtained from UAV imagery compared to traditional ground counts to determine what bias may exist as a result of behavioral responses to UAV flights.

## Supporting information

S1 Table S2 provides statistical tables not included in the main manuscript. (DOCX)

S1 File S1 Appendix provides details on UAV system, field operations, sensor and data collection, regulations, logistics and any permits obtained for the project. (DOCX)

S2 File S3 Data breeding dabbling duck behavior data for focal surveys. (CSV)

S3 File S4 Data breeding dabbling duck data for scan surveys including metadata. (XLSX)

## References

[1] Sasse DB. Job-related mortality of wildlife workers in the United States, 1937–2000. Wildlife Society Bulletin. 2003;1015–20.

[2] George Pierce Jones Pearlstine LG, Percival HF. An Assessment of Small Unmanned Aerial Vehicles for Wildlife Research. Wildlife Society Bulletin. 2006;34(3):750–8.

[3] Weissensteiner MH, Poelstra JW, Wolf JB. Low‐budget ready‐to‐fly unmanned aerial vehicles: An effective tool for evaluating the nesting status of canopy‐breeding bird species. Journal of avian biology. 2015;46(4):425–30.

[4] Anderson K, Gaston KJ. Lightweight unmanned aerial vehicles will revolutionize spatial ecology. Frontiers in Ecology and the Environment. 2013;11(3):138–46.

[5] Jones GP. The feasibility of using small unmanned aerial vehicles for wildlife research [PhD Thesis]. University of Florida USA; 2003.

[6] Linchant J, Lisein J, Semeki J, Lejeune P, Vermeulen C. Are unmanned aircraft systems (UAS s) the future of wildlife monitoring? A review of accomplishments and challenges. Mammal Review. 2015;45(4):239–52.

[7] Christie KS, Gilbert SL, Brown CL, Hatfield M, Hanson L. Unmanned aircraft systems in wildlife research: current and future applications of a transformative technology. Frontiers in Ecology and the Environment. 2016;14(5):241–51.

[8] Barnas A, Newman R, Felege CJ, Corcoran MP, Hervey SD, Stechmann TJ, et al. Evaluating behavioral responses of nesting lesser snow geese to unmanned aircraft surveys. Ecology and evolution. 2018;8(2):1328–38. doi: 10.1002/ece3.3731 29375801 PMC5773326

[9] McEvoy JF, Hall GP, McDonald PG. Evaluation of unmanned aerial vehicle shape, flight path and camera type for waterfowl surveys: disturbance effects and species recognition. PeerJ. 2016;4:e1831. doi: 10.7717/peerj.1831 27020132 PMC4806640

[10] Vas E, Lescroël A, Duriez O, Boguszewski G, Grémillet D. Approaching birds with drones: first experiments and ethical guidelines. Biology letters. 2015;11(2):20140754. doi: 10.1098/rsbl.2014.0754 25652220 PMC4360097

[11] Mulero-Pázmány M, Jenni-Eiermann S, Strebel N, Sattler T, Negro JJ, Tablado Z. Unmanned aircraft systems as a new source of disturbance for wildlife: A systematic review. PloS one. 2017;12(6):e0178448. doi: 10.1371/journal.pone.0178448 28636611 PMC5479521

[12] Frid A, Dill L. Human-caused disturbance stimuli as a form of predation risk. Conservation Ecology. 2002;6(1).

[13] Ditmer MA, Vincent JB, Werden LK, Tanner JC, Laske TG, Iaizzo PA, et al. Bears show a physiological but limited behavioral response to unmanned aerial vehicles. Current Biology. 2015;25(17):2278–83. doi: 10.1016/j.cub.2015.07.024 26279232

[14] Brisson-Curadeau É, Bird D, Burke C, Fifield DA, Pace P, Sherley RB, et al. Seabird species vary in behavioural response to drone census. Scientific reports. 2017;7(1):17884. doi: 10.1038/s41598-017-18202-3 29263372 PMC5738335

[15] Dulava S, Bean WT, Richmond OM. Environmental reviews and case studies: applications of unmanned aircraft systems (UAS) for waterbird surveys. Environmental Practice. 2015;17(03):201–10.

[16] Bushaw JD, Ringelman KM, Johnson MK, Rohrer T, Rohwer FC. Applications of an unmanned aerial vehicle and thermal-imaging camera to study ducks nesting over water. Journal of Field Ornithology. 2020;91(4):409–20.

[17] Pöysä H, Kotilainen J, Väänänen V-M, Kunnasranta M. Estimating production in ducks: a comparison between ground surveys and unmanned aircraft surveys. European journal of wildlife research. 2018;64(6):74.

[18] Drever MC, Chabot D, O’Hara PD, Thomas JD, Breault A, Millikin RL. Evaluation of an unmanned rotorcraft to monitor wintering waterbirds and coastal habitats in British Columbia, Canada. Journal of Unmanned Vehicle Systems. 2015;3(4):256–67.

[19] Dundas SJ, Vardanega M, O’Brien P, McLeod SR. Quantifying Waterfowl Numbers: Comparison of Drone and Ground-Based Survey Methods for Surveying Waterfowl on Artificial Waterbodies. Drones. 2021 Mar;5(1):5.

[20] Jarrett D, Calladine J, Cotton A, Wilson MW, Humphreys E. Behavioural responses of non-breeding waterbirds to drone approach are associated with flock size and habitat. Bird Study. 2020;67(2):190–6.

[21] Altmann J. Observational study of behavior: sampling methods. Behaviour. 1974;49(3–4):227–66. doi: 10.1163/156853974x00534 4597405

[22] Stewart RE, Kantrud HA. Classification of natural ponds and lakes in the glaciated prairie region. Vol. 92. US Bureau of Sport Fisheries and Wildlife; 1971.

[23] Rischette AC, Hovick TJ, Elmore RD, Geaumont BA. Use of small unmanned aerial systems for sharp-tailed grouse lek surveys. Wildlife Biology. 2020;2020(2).

[24] Burnham KP, Anderson DR. A practical information-theoretic approach. Model selection and multimodel inference, 2nd ed Springer, New York. 2002;2.

[25] Rümmler M-C, Mustafa O, Maercker J, Peter H-U, Esefeld J. Measuring the influence of unmanned aerial vehicles on Adélie penguins. Polar Biology. 2015;39(7):1329–34.

[26] Weimerskirch H, Prudor A, Schull Q. Flights of drones over sub-Antarctic seabirds show species-and status-specific behavioural and physiological responses. Polar Biology. 2018;41(2):259–66.

[27] Egan CC, Blackwell BF, Fernández-Juricic E, Klug PE. Testing a key assumption of using drones as frightening devices: Do birds perceive drones as risky? The Condor. 2020. doi: 10.1093/condor/duaa009 32476673 PMC7243448

[28] Ellis-Felege SN, Stechmann TJ, Hervey SD, Felege CJ, Rockwell RF, Barnas AF. Nesting common eiders (Somateria mollissima) show little behavioral response to fixed-wing drone surveys. J Unmanned Veh Sys [Internet]. 2021 Oct 28 [cited 2021 Nov 30]; Available from: https://cdnsciencepub.com/doi/abs/ doi: 10.1139/juvs-2021-0012

[29] Bélanger L, Bédard J. Energetic Cost of Man-Induced Disturbance to Staging Snow Geese. The Journal of Wildlife Management. 1990;54(1):36–41.

[30] Duffy J, Shapiro A, Anderson K, Spina Avino F, DeBell L, Glover-Kapfer P. Conservation Technology Series Issue 5: DRONES FOR CONSERVATION. 2020.

[31] Tabak MA, Norouzzadeh MS, Wolfson DW, Sweeney SJ, Vercauteren KC, Snow NP, et al. Machine learning to classify animal species in camera trap images: Applications in ecology. Methods in Ecology and Evolution. 2019;10(4):585–90.

[32] Helvey M. Application of Thermal and Ultraviolet Sensors in Remote Sensing of Upland Ducks. Theses [Internet]. 2020 Jul 28; Available from: https://scholarworks.rit.edu/theses/10536

