# Supplementary material for: Behavioral responses of blue-winged teal and northern shoveler to unmanned aerial vehicle surveys
Source: PLoS One. 2022 Jan 19;17(1):e0262393. doi: 10.1371/journal.pone.0262393 (PMC8769346; doi:10.1371/journal.pone.0262393)
Supplement: S1 Table — (DOCX) [file pone.0262393.s001.docx]

**Supplement 2 – Full statistical output from modeling**

**Supplement 1a: Estimates (model scale linear predictor), expected values (μ, data scale) and 95% confidence intervals from the model for the 3-way interaction (species x treatment x flight period) describing the proportion of time spent on behaviors of blue-winged teal and northern shovelers during UAV flight to treatment, and flight period**. Estimates obtained from 151 observations across 32 flights for blue-winged teal, and 46 observations across 13 flights for northern shovelers.

| **Blue-winged teal** |  | | | | | |
| --- | --- | --- | --- | --- | --- | --- |
| **Behavior** | **CTRL x BEFORE** | **CTRL x DURING** | **CTRL x AFTER** | **UAV x BEFORE** | **UAV x DURING** | **UAV x AFTER** |
| **Active** |  |  |  |  |  |  |
| Estimate | 1.1712 | 1.6956 | 1.3791 | 2.1683 | 1.6149 | 2.1703 |
| μ | 0.7634 | 0.8450 | 0.7988 | 0.8974 | 0.8341 | 0.8975 |
| 95% CI | 0.6425 < μ < 0.8527 | 0.7459 < μ < 0.9100 | 0.6882 < μ < 0.8772 | 0.8202 < μ < 0.9437 | 0.7246 < μ < 0.9057 | 0.8182 < μ < 0.9446 |
| **None** |  |  |  |  |  |  |
| Estimate | -1.2908 | -1.7683 | -1.5226 | -2.3195 | -1.8223 | -2.3230 |
| μ | 0.2157 | 0.1458 | 0.1791 | 0.0895 | 0.1392 | 0.0892 |
| 95% CI | 0.1288 < μ < 0.3385 | 0.0817 < μ < 0.2465 | 0.1048 < μ < 0.2891 | 0.0469 < μ < 0.1643 | 0.0749 < μ < 0.2439 | 0.0461 < μ < 0.1658 |
| **Vigilant** |  |  |  |  |  |  |
| Estimate | -4.4400 | -4.8338 | -4.4456 | -4.4415 | -4.7999 | -4.4253 |
| μ | 0.0117 | 0.0079 | 0.0116 | 0.0116 | 0.0082 | 0.0118 |
| 95% CI | 0.0096 < μ < 0.0142 | 0.0063 < μ < 0.0099 | 0.0095 < μ < 0.0141 | 0.0096 < μ < 0.0142 | 0.0065 < μ < 0.0103 | 0.0096 < μ < 0.0145 |
| **Overhead Vigilance** |  |  |  |  |  |  |
| Estimate | -4.5789 | -4.7079 | -4.7560 | -4.8320 | -3.9161 | -4.8610 |
| μ | 0.0102 | 0.0089 | 0.0085 | 0.0079 | 0.0195 | 0.0077 |
| 95% CI | 0.0083 < μ < 0.0124 | 0.0072 < μ < 0.0110 | 0.0070 < μ < 0.0105 | 0.0064 < μ < 0.0098 | 0.0163 < μ < 0.0233 | 0.0062 < μ < 0.0096 |
| **Northern Shoveler** |  | | | | | |
|  |  |  |  |  |  |  |
| **Active** |  |  |  |  |  |  |
| Estimate | 3.1664 | 2.2356 | 0.1833 | 0.9649 | 2.2635 | 1.6717 |
| μ | 0.9596 | 0.9034 | 0.5457 | 0.7241 | 0.9058 | 0.8418 |
| 95% CI | 0.8860 < μ < 0.9864 | 0.7580 < μ < 0.9654 | 0.3065 < μ < 0.7655 | 0.5056 < μ < 0.8707 | 0.7756 < μ < 0.9640 | 0.6610 < μ < 0.9356 |
| **None** |  |  |  |  |  |  |
| Estimate | -3.3231 | -2.2549 | -0.0696 | -0.9866 | -2.8368 | -1.8393 |
| μ | 0.0348 | 0.0950 | 0.4826 | 0.2716 | 0.0554 | 0.1371 |
| 95% CI | 0.0107 < μ < 0.1072 | 0.0317 < μ < 0.2515 | 0.2452 < μ < 0.7281 | 0.1217 < μ < 0.5009 | 0.0186 < μ < 0.1537 | 0.0524 < μ < 0.3134 |
| **Vigilant** |  |  |  |  |  |  |
| Estimate | -4.5577 | -4.3802 | -4.5989 | -4.9501 | -4.0148 | -4.5153 |
| μ | 0.0104 | 0.0124 | 0.0100 | 0.0070 | 0.0177 | 0.0108 |
| 95% CI | 0.0074 < μ < 0.0145 | 0.0087 < μ < 0.0175 | 0.0065 < μ < 0.0153 | 0.0046 < μ < 0.0106 | 0.0127 < μ < 0.0246 | 0.0073 < μ < 0.0160 |
| **Overhead Vigilance** |  |  |  |  |  |  |
| Estimate | -4.4749 | -4.9971 | -5.1104 | -5.0970 | -3.4256 | -4.4301 |
| μ | 0.0113 | 0.0067 | 0.0060 | 0.0061 | 0.0315 | 0.0118 |
| 95% CI | 0.0085 < μ < 0.0149 | 0.0047 < μ < 0.0096 | 0.0040 < μ < 0.0089 | 0.0042 < μ < 0.0088 | 0.0242 < μ < 0.0409 | 0.0084 < μ < 0.0166 |

**Supplement 1b:Model selection results for ducks swimming away from UAV or towards cover during UAV surveys based on scan surveys conducted at flight and control wetlands**.

| **Model** | **AICc** | **∆AIC** | **Weight** | **-2LogLikelihood** |
| --- | --- | --- | --- | --- |
| Intercept + Distance From Pad | 86.95 | 0 | 0.98859 | 82.77 |
| Intercept + Treatment | 96.44 | 9.49 | 0.00860 | 92.29 |
| Intercept + Treatment + Cover Type + Plot Coverage + Distance From Pad + Wind Speed +Wind Direction+ Temperature + Cloud Cover | 99.89 | 12.94 | 0.00153 | 49.34 |
| Intercept + Plot Coverage | 102.26 | 15.31 | 0.00047 | 98.11 |
| Intercept + Treatment + Wind Speed + Wind Direction | 102.91 | 15.96 | 0.00034 | 79.72 |
| Intercept only | 104.33 | 17.38 | 0.00017 | 102.28 |
| Intercept + Cloud Cover | 105.23 | 18.28 | 0.00011 | 96.73 |
| Intercept + Temperature | 105.87 | 18.92 | 0.00008 | 101.72 |
| Intercept + Wind Speed | 106.34 | 19.39 | 0.00006 | 102.19 |
| Intercept + Wind direction | 106.85 | 19.9 | 0.00005 | 88.83 |
| Intercept + Cover Type | 108.32 | 21.37 | 0.00002 | 102.02 |
