# Supplementary material for: Behavioral responses of blue-winged teal and northern shoveler to unmanned aerial vehicle surveys
Source: PLoS One. 2022 Jan 19;17(1):e0262393. doi: 10.1371/journal.pone.0262393 (PMC8769346; doi:10.1371/journal.pone.0262393)
Supplement: S1 File — (DOCX) [file pone.0262393.s002.docx]

**Supplementary Data**

UAV Reporting Protocol Example

Produced by: Mason Ryckman and Susan Ellis-Felege, April 2021

Citation:

1. **Project Overview**

The objective of this research was to examine the behavioral impact of Unmanned Aerial Vehicle (UAV) surveys on blue-winged teal (*Spatula discors*) and northern shovelers (*Spatula clypeata*) during spring when pair surveys are conducted to estimate potential productivity. Using GoPros attached to spotting scopes, we examined behavioral responses of ducks using focal surveys on wetlands that were flown over and compared them to ducks that were not flown over. We also examined the number of flushes across three flight periods (pre-, during, and post-UAV flight). We determined the best predictor for ducks swimming away or towards cover during flights based upon scan surveys during the flight. For the purpose of this study, we did not use imagery collected from the UAV, but sensor specifications are described in section 3 as they dictated the flight details such as altitude.

1. **UAV System and Operation Details**

*2.1 Platform specifications*

Flights were conducted with a quad-rotary, DJI Matrice 200 v2 (Figure 1). Aircraft specifications are as follows; color: black, weight: 4.69kg, operating temp: -20°C to 50°C, power source: removable lithium polymer batteries (22.8V, 7660 mAh), estimated maximum endurance: 38min. For specifications of latest available models see: <https://www.dji.com/matrice-200-series-v2/info>


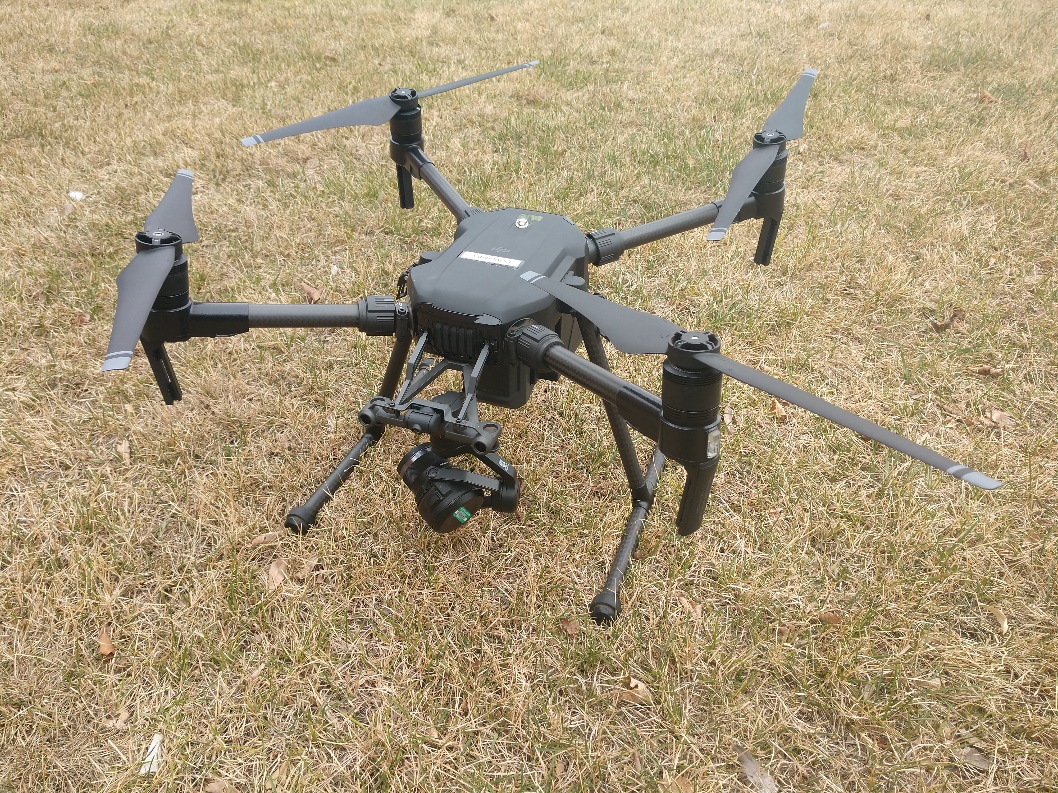


**Fig. 1. DJI Matrice 200 v2**

*2.2 Flight planning and method of operation*

Matrice 200 v2 uses a vertical takeoff and landing method which is initiated manually by the UAV operator. All flight plans were preprogrammed autonomous line transects using DJI Pilot (version 1.8.0). We used a DJI CrystalSky monitor (7.85in, ultra-bright) attached to the UAV controller to plan flights and monitor the UAVs position in real time. Flight planning consisted of specifying the boundary points and choosing an image overlap (60% forward and side) which determined the distance between transects (Figure 2). Start and end points were arranged so the end point was closer to the launch site. The UAV operator would manually take-off and ascend to approximately 45m above ground level (AGL) before starting the autonomous flight path. All flights were flown at 45m which allowed us the ability identify ducks to species and sex using our current sensor. During flight mission, the Matrice 200 executed flight plans automatically, but the UAV operator had the ability for safety interventions such as pausing the mission and manually return the aircraft back to the launch site or home pad.


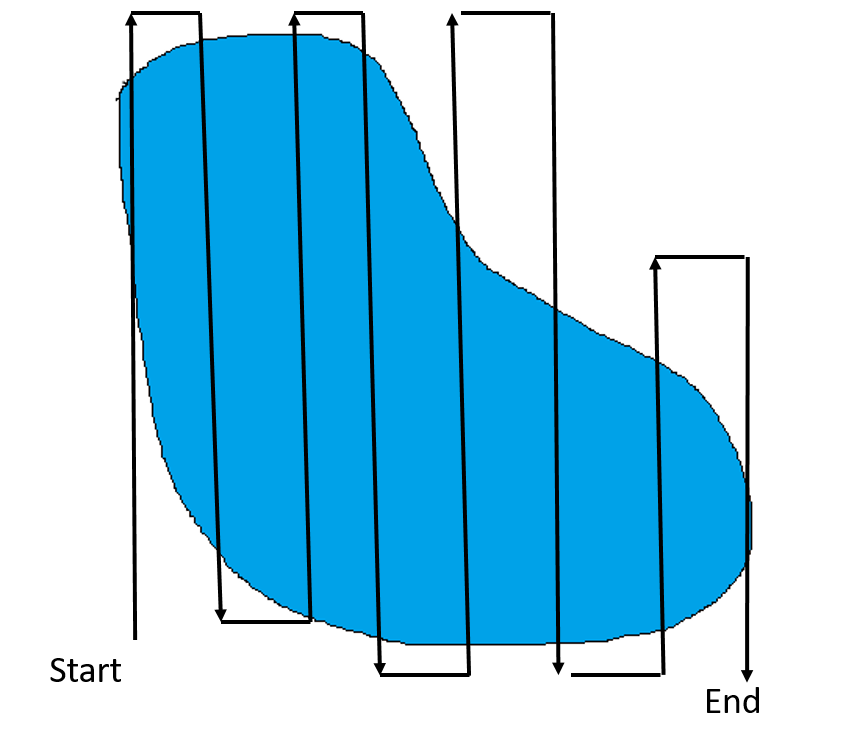


**Figure 2. Example Matrice 200 flight lines at a wetland showing the starting point, “lawn mower” transects, and ending point of the survey.**

1. **Sensor and Data Collection**

The nature of this study involved the behavioral reaction of UAV flights, but our goal was to evaluate behaviors as if we were conducting a survey that would allow for identification of ducks to sex and species. As a result, the flight protocols were based upon sensor limitations. A Zenmuse X5S RGB camera with an Olympus 45mm lens was used as our sensor. At 45m altitude, the sensor has a ground sampling distance of roughly 0.44cm per pixel. We used a 60% front and side overlap between images to allow orthomosaics to be produced for a future project conducting counts. Images were stored on two micro-SD cards onboard the drone and downloaded onto hard-drives at the end of each survey day. They were later uploaded and are stored on the UND High Performance Computational Center servers.

1. **Field Operation Details**

All flights were conducted with the same rotary platform. In total we conducted 42 flight

and collected behavioral data from 151 blue-winged teal and 46 northern shovelers (Table 1). Average flight duration was 18 min (range: 6-29 minutes). Environmental conditions such as wind speed and temperature were measured at the beginning and end of each flight and averaged to obtain one value. Both were recorded using a Kestrel 3000 (Global Test Supply). Average wind speed and temperature for flights was 8.70km/ h (range: 0-19.31km h^-1^) and 17.32°C (range: 2.53-26.42°C). During field operations, we did not need to execute any UAV flights for safety intervention procedures.

**Table 1. Summary of UAV flights used in behavioral analyses of breeding dabbling ducks.**

| **Flight Date (M/D/Y)** | **Wind Speed (km/h)** | **Temperature (°C)** | **Flight Duration (mins)** |
| --- | --- | --- | --- |
| 04/24/2020 | 1.13 | 18.78 | 16 |
| 04/24/2020 | 5.15 | 18.67 | 25 |
| 04/25/2020 | 8.45 | 11.75 | 10 |
| 04/25/2020 | 9.66 | 14.22 | 20 |
| 04/27/2020 | 0 | 14.17 | 12 |
| 04/27/2020 | 0 | 21.58 | 23 |
| 04/27/2020 | 4.02 | 20.64 | 9 |
| 04/29/2020 | 8.53 | 11.33 | 23 |
| 04/29/2020 | 9.33 | 13.42 | 13 |
| 04/29/2020 | 4.18 | 18.61 | 13 |
| 05/03/2020 | 14.24 | 8.06 | 7 |
| 05/03/2020 | 19.23 | 12.89 | 24 |
| 05/03/2020 | 18.75 | 14.33 | 23 |
| 05/06/2020 | 6.68 | 11 | 11 |
| 05/06/2020 | 4.83 | 17.58 | 21 |
| 05/06/2020 | 3.86 | 19 | 15 |
| 05/08/2020 | 5.71 | 7.17 | 11 |
| 05/08/2020 | 4.02 | 15.64 | 14 |
| 05/08/2020 | 7.40 | 17.17 | 24 |
| 05/11/2020 | 8.29 | 2.53 | 15 |
| 05/11/2020 | 5.71 | 6.36 | 17 |
| 05/11/2020 | 11.59 | 10.17 | 11 |
| 05/11/2020 | 6.20 | 8.75 | 16 |
| 05/15/2020 | 4.02 | 17.53 | 13 |
| 05/15/2020 | 7.24 | 21.53 | 13 |
| 05/15/2020 | 6.04 | 20.03 | 18 |
| 05/15/2020 | 11.27 | 20.92 | 13 |
| 05/16/2020 | 9.09 | 17.08 | 26 |
| 05/16/2020 | 7.64 | 19.39 | 27 |
| 05/16/2020 | 13.44 | 19.69 | 29 |
| 05/22/2020 | 12.39 | 18.44 | 24 |
| 05/22/2020 | 14.24 | 24.83 | 16 |
| 05/22/2020 | 18.11 | 25.36 | 20 |
| 05/23/2020 | 9.09 | 24.92 | 26 |
| 05/23/2020 | 3.14 | 26.42 | 25 |
| 05/26/2020 | 6.68 | 16.69 | 28 |
| 05/26/2020 | 6.60 | 22.5 | 26 |
| 05/26/2020 | 6.36 | 23.22 | 26 |
| 05/26/2020 | 12.87 | 23.69 | 23 |
| 05/27/2020 | 14.48 | 19.56 | 14 |
| 05/27/2020 | 16.58 | 26.31 | 23 |
| 05/27/2020 | 19.31 | 25.5 | 20 |

1. **Data Post-Processing**

Not applicable, see section 3.

1. **Permits, Regulations, and Logistics**

Operation of the Matrice 200 was contingent on the operators obtaining a small unmanned aircraft system remote pilot license (Part 107 FAA Operator License). UAV flights and data collection on breeding dabbling ducks were permitted by the University of North Dakota Institutional Animal Care and Use Committee approvals A3917-01, Protocol #1904-2. The UND Unmanned Aircraft System Research Compliance Committee reviewed human privacy and data management protocols for the project (Approved April 12, 2019), and permissions were provided by the North Dakota Game and Fish Department (GNF04912726, GNF05182785). Flights were conducted in collaboration with Ducks Unlimited, Inc and The Nature Conservancy that provided site access for the project.

All UAV flights followed FAA rules for operating small unmanned aerial vehicles to include operating within visual line of sight at all times, during daylight hours, and below 122 m or 400 ft AGL. Further all flights occurred with in class G airspace. Most flights launch sites or home pad locations were in proximity to a gravel road or prairie trail, and we did not experience any logistic difficulties with transportation of the aircraft.
